# A faculty-wide “Night of Skills” to (not only) train medical skills: comprehensive evaluation study

**DOI:** 10.1186/s12909-026-09066-1

**Published:** 2026-05-09

**Authors:** Moritz Mahling, Thomas Shiozawa, Lea Herschbach, Tobias Albrecht, Tim Schöne, Jan Griewatz, Bernhard Hirt, Stephan Zipfel, Anne Herrmann-Werner, Friederike Holderried

**Affiliations:** 1 Department of Medical Strategy, Process and Quality Management, University Hospital Tübingen, Tübingen, Germany; 2 Department of Anatomy, Institute of Clinical Anatomy and Cell Analysis, Faculty of Medicine, Eberhard Karls University of Tübingen, Tübingen, Germany; 3 Tübingen Institute for Medical Education (TIME), Eberhard Karls University, Tübingen, Germany; 4 Faculty of Medicine, University of Tübingen, Tübingen, Germany; 5 Department of Psychosomatic Medicine and Psychotherapy, University Hospital Tübingen, Tübingen, Germany

**Keywords:** Education, medical, undergraduate, Clinical competence, Simulation training, Curriculum, Self efficacy, Extracurricular activities, Medical skills training, Interprofessional education

## Abstract

**Background:**

This study evaluates the educational and social impact of a faculty-wide “Night of Skills” event for health professions students. The event combined short, hands-on medical skills workshops with opportunities for informal exchange and networking across disciplines.

**Methods:**

The event ran on a Friday from 5 pm to 3 am, with 35 workshops across medical disciplines with 332 workshop timeslots (30 min each). The social program included information and networking booths, as well as food, drinks and a closing event. Feedback was collected with semi-structured oral interviews and a survey, collecting participant demographics and feedback using Likert-scaled questions and open questions. Quantitative data were analyzed using descriptive statistics, while qualitative responses underwent thematic analysis based on Mayring’s inductive category formation.

**Results:**

A total of 396 students participated, mostly from human medicine (91.2%, *N* = 361). We recorded 1524 workshop bookings. Most participants attended an average of five workshops and expressed a desire for additional sessions. The timeslots were seen as too short, but most participants (83.6%, *N* = 143) rated their learning outcome as “high” or “very high.” Participants reported increased self-efficacy and preparedness for clinical clerkships. Networking opportunities and confidence building were highlighted in qualitative feedback. Of 189 respondents, 96.3% (*N* = 182) rated the event “very good” or “good”, with 98.4% (*N* = 186) wanting it to be repeated.

**Conclusions:**

The “Night of Skills” demonstrated high perceived educational and social value among participants. It offers a prototype for supplementing medical curricula with practice-oriented, community-building experiences that can be adapted by other institutions. Future studies incorporating objective skill assessments are needed to confirm the educational benefits suggested by these self-reported outcomes.

**Clinical trial number:**

Not applicable

**Supplementary Information:**

The online version contains supplementary material available at 10.1186/s12909-026-09066-1.

## Background

A primary goal of health professions education is to ensure that graduates are equipped with the skills and competency to deliver safe and effective patient care [1]. Competency-based medical education (CBME) supports this objective [2–5], though the precise definitions of “competency” continue to be subject of ongoing discussion [6, 7]. Within CBME frameworks, “skills” are considered a key element of many competencies [4, 5, 8]. Regarding the definition of “skill”, Benjamin Bloom’s influential taxonomy refers to the “psychomotor domain” or “manipulative or motor-skill area” [9], and a widely used educational definition (“the mental and motor activities required to execute manual tasks” [10]) also emphasizes manual activities more explicitly for “skills” compared to the broader term “competency.”

Historically, students in health professions gained skills by working with real patients in clinical settings [11]. However, growing educational demands, patient safety concerns, and technological advancements challenged this approach [12]. By the 1970 s, skill training began shifting to dedicated Clinical Skills Laboratories (Skills Labs) [12, 13]. These allow for consistent training, providing scheduled access to patient simulators and skilled instructors [11, 14]. Since their inception, the efficacy of medical skills labs has been demonstrated [15], with key factors such as “providing feedback” and “repetitive practice” identified as essential for successful learning outcomes—both central components of deliberate practice [16–18].

The contact restrictions introduced by COVID-19 highlighted an important, previously less emphasized factor: the necessity of being physically present at the place of learning. With strict contact restrictions, clinical electives and face-to-face teaching were reduced or cancelled altogether [19–21]. These changes not only caused considerable distress among medical students [22], but also posed significant challenges to health professions education [23]. It can be assumed that these disruptions had negative impacts on health professions students [24].

Reflecting these global challenges, medical students at our institution expressed similar concerns, despite the faculty’s maximum efforts to mitigate the limitations. In a faculty meeting, a “Night of Skills” has been proposed. This event, intended to take place over one evening, should offer a range of practical learning opportunities for interested students. Moreover, it would symbolize a return to in-person teaching and provide an opportunity to reconnect face-to-face. However, there were concerns about the educational value of a one-time skills training event, particularly since it would not be directly integrated into the curriculum for most participants. From the perspective of deliberate practice, this critique is understandable [17]. Furthermore, it is well known that distributed training sessions are superior to one time, massed training in terms of skill retention [25, 26].

Nonetheless, the faculty believed that the event would serve additional purposes beyond skill development, and thus, decided to proceed with the “Night of Skills”. Given the paucity of published work on comparable, faculty-wide extracurricular skills events, the concept was developed locally. This decision was consistent with the faculty’s commitment to foster extracurricular educational activities, which also have been shown to improve students’ motivation, academic skills and professional development beyond specific situations such as the COVID-19 pandemic [27]. The event was conceptually aligned with principles of experiential learning [28, 29] and communities of practice [30], assuming that even a one‑time, practice‑oriented exposure to skills training and faculty interaction could support learning and professional socialization. Principles of competency-based medical education and deliberate practice served as a reference framework to contextualize skill-related learning objectives, while acknowledging the limitations of a one-time, non-curricular format.

As it was important to gain deeper insights into the students’ motivations and perceived benefits of such an event. Therefore, it was decided to conduct a thorough evaluation, using both oral interviews during the event and an online survey afterwards. The evaluation was guided by the research question “How do participants of a faculty‑wide “Night of Skills” event evaluate the organizational framework and the perceived educational value of the event?“ In this article, a brief overview of the event itself and the findings of our evaluation are presented.

## Methods

### Description of the event

The “Night of Skills” was organized by faculty and student groups and took place in June 2023. Event locations were geographically close to ensure short walks, and the event ran on a Friday from 5 pm to 3 am. The event was announced via the faculties’ communication channels, and students registered through an online system, booking a “campus ticket” for access. They could also reserve up to five workshop timeslots on a first-come, first-served basis.

From 5 pm to 1 am, 35 workshops (Table 1) were offered, each lasting 30 min with 15 min for changeover. The number of participants per workshop ranged from 1 to 10 (mean ± standard deviation: 5.3 ± 2.9), depending on prerequisites. Not all workshops were available for all 10 timeslots, resulting in 332 bookable sessions. These were hosted by the Medical Faculty and University Hospital Tübingen, staffed by healthcare professionals and student tutors. Workshops were offered in German and English, and had defined target groups and descriptions visible in the registration system.

**Table 1:** List of all 35 workshops offered at the “Night of Skills” (2 workshops offered 2 times, indicated by “2x”)

Advanced medical communication skills	Airway management
Aseptic dressing change	Arterial blood gas sampling and thoracentesis
Basic medical communication skills	Bronchoscopy simulator training
Cardiopulmonary Resuscitation (CPR)	Central venous catheterization (CVC) on donor specimens
Central venous catheterization (CVC) on simulators	Clinical shoulder examination techniques
Cricothyroidotomy training	Emergency radiology interpretation
Endoscopic anatomy on donor specimens	Epithetic care in otolaryngology
Fetal rotation during labor and delivery	Functional endoscopic sinus surgery
Head and neck ultrasonography	Histological microscopy techniques
Laparoscopic skills training	Laparoscopy on donor specimens (**2x**)
Newborn physical examination	Oropharyngeal and endotracheal suctioning
Pediatric basic life support	Pediatric emergency simulation training
Petrous bone dissection exercises	Point-of-care emergency ultrasonography
Prostate pathology assessment and diagnosis	Surgical abdominal examination
Suturing techniques workshop	Trauma management
Transurethral resection (TUR) simulator training	Venipuncture and peripheral IV cannulation (**2x**)
Virtual reality medical simulation	

A social program fostered exchange between students and lecturers, including a recorded dean’s speech, information booths, music, food, and non-alcoholic drinks. Two closing events took place from 1 am to 3 am. Due to the large amount of expected participants, the safety of participants was ensured by security service personnel.

### Evaluation of the event

To gain a detailed understanding of the potential benefits of the event, an in-depth evaluation using data from three different perspectives was planned, specified in the following. This study used a mixed-methods evaluation approach including registration data analysis, interviews and surveys. The only inclusion criterion was willingness to participate in the evaluation, and the only exclusion criteria was inadequate language skills.

#### Online registration system (before the event)

All students participating in workshops and all student tutors were required to register upfront via an online registration system (pretix, rami.io GmbH, Heidelberg, Germany). During this process, they were asked to provide gender, age, study program, and their role (participant or tutor). Faculty members, staff, and helpers involved in the organization or supervision of the event were not required to register and were therefore not included in the registration dataset.

#### Oral interviews (during the event)

To gain a deeper understanding of the students’ assessment, interviews were conducted verbally during the event using an interview guide that covered aspects such as motivation, expectations, benefits from the event and further wishes (**Additional File 1**). The audio was recorded and subsequently transcribed using the software Sonix.ai (Sonix, Inc., California). Participation was voluntary, with informed consent obtained verbally prior to each interview.

#### Written evaluation

After the event, all participants were emailed to voluntarily complete a digital survey via the institution’s approved evaluation system, EvaSys (evasys GmbH, Germany). Though primarily used for course evaluation, the anonymous data was also used for this research. The questionnaire covered demographics, event aspects, detailed workshop evaluations, and feedback on the social program. Open text questions asked for workshop suggestions and general feedback on workshops, the social program, and the event. The survey items were developed by the study team based on existing internal evaluation instruments and refined through expert discussion within the organizing committee. Given the descriptive and evaluative focus of the study, no formal psychometric validation was conducted.

### Analysis of data

#### Qualitative analysis

To gain a deeper understanding of the qualitative analysis, data from oral interviews and the written evaluation was combined. Categories were created using Mayring’s inductive category formation, ensuring the analysis is grounded in participants’ responses [31]. The oral interviews were distilled to essentials using the interview guide, aiming to identify categories that highlight participant differences and distinct experiences. Student responses from the written evaluation were classified into the same categories (Table 2).

**Table 2:** Categories for the qualitative analysis

Categories for qualitative analysis of the interviews
Motivation
Expectations
Expectations met
New skills aquired
New contacts established
Benefited in other ways
Preparation for clinic
Self-efficacy
Improvement ideas organisation and framework
Improvement ideas workshops
Positive feedback/requests
**Additional categories from the written evaluation**
Subjects that should be offered again
Subjects that should not be offered again
Social program and framework that should be offered again
Social program and framework that should not be offered again

#### Quantitative Analysis

Quantitative data are presented as mean ± standard deviation or, for non-Gaussian distributions, as median with 25%/75% quartiles (Q25 – Q75). Likert-scale questionnaire results are shown in centred Likert plots. Given the exploratory and evaluative nature of the study, no inferential statistical tests or group comparisons were performed. The analysis was conducted using R (version 4.3.1, The R Foundation for Statistical Computing, Vienna, Austria). The sample represent a convenience sample without sample size calculation.

### Ethical approval

The evaluation followed the Declaration of Helsinki. Participation in the event and data collection were voluntary. While face-to-face anonymity wasn’t possible during interviews, recordings, transcripts, and registration data were anonymized. Written evaluations were fully anonymous. As a strictly anonymous, low-risk educational evaluation, no written informed consent was required according to local regulations. Participants were informed verbally, and consent was obtained verbally for interviews; survey completion was considered implied consent. The study plan has been submitted to the Medical Faculty of the University of Tübingen’s ethical committee (Nr. 362/2023BO2) with no objections raised.

## Results

### Description of participants

The detailed characteristics of the participants are displayed in Table 3. Detailed and comprehensive numeric questionnaire data are available as Additional File 2 of the 396 total attendees (including 294 participants and 102 tutors), 189 completed the written evaluation, resulting in an overall response rate of 47.7%. 

**Table 3:** Detailed characteristics of the participants from the registration system, the oral interviews and the written evaluation. ^1^Only participating students and tutors registered for the event, faculty and other staff did not need to register for the event and are therefore not included in the registration dataset

Characteristic	Registration system (upfront)		Oral Interviews (during event)		Written Evaluation (after event)	
	Median (Q25 – Q75)				Median (Q25 – Q75)	
**Age**	23 (21–25)		-	-	22 (21–25)	
	**N**	**%**	**N**	**%**	**N**	**%**
**N total**	396	100.0%	27	100.0%	189	100.0%
**Gender**						
Female	276	69.7%	22	81.5	131	69.3%
Male	118	29.8%	5	18.5	53	28.0%
Diverse	2	0.51%	0	0	2	1.1%
NA	0	0.0%	0	0	3	1.6%
**Faculty**						
Human Medicine	361	91.2%	25	92.6%	171	90.5%
Medical technology	9	2.3%	0	0.0%	3	1.6%
Midwifery	14	3.5%	0	0.0%	5	2.6%
Molecular Medicine	1	0.3%	0	0.0%	0	0.0%
Neurosciences	2	0.5%	0	0.0%	0	0.0%
Nursing	9	2.3%	1	3.7%	5	2.6%
Other/NA	0	0.0%	1	3.7%	5	2.6%
**Role**						
Participant	294	74.2%	19	70.4%	173	91.5%
Tutor	102	25.8%	4	14.8%	7	3.7%
Faculty/Staff/Helper	-^1^	-^1^	3	11.1%	9	4.8%
Simulation person	-^1^	-^1^	1	3.7	0	0.0%

### Organisation and timeframe

#### Source of registration

**Fig. 1:**
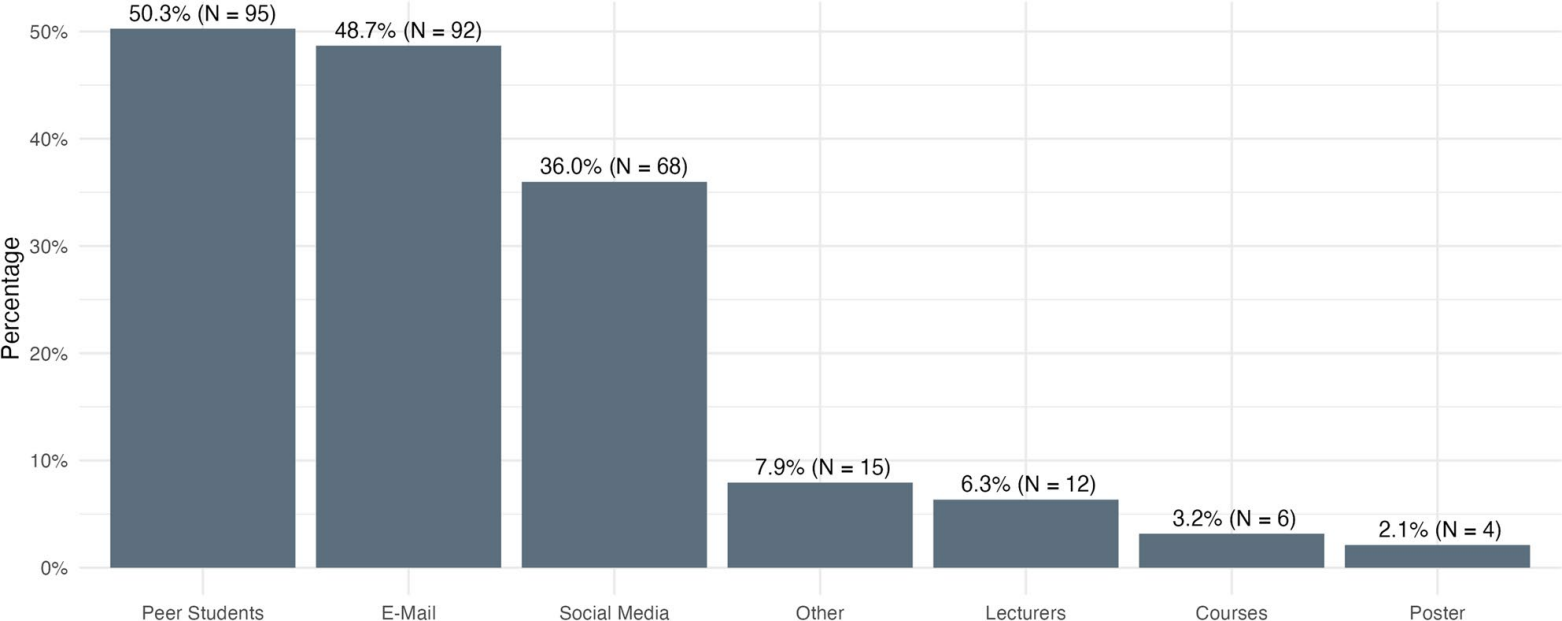
Source of information for registration as stated by the participants in the written evaluation

During the written evaluation, 189 participants made 292 selections about their source of information for registration (Fig. 1). Peer students and E-Mail were the primary sources, followed closely by social media.

#### Quantitative feedback on timeframe

**Fig. 2:**
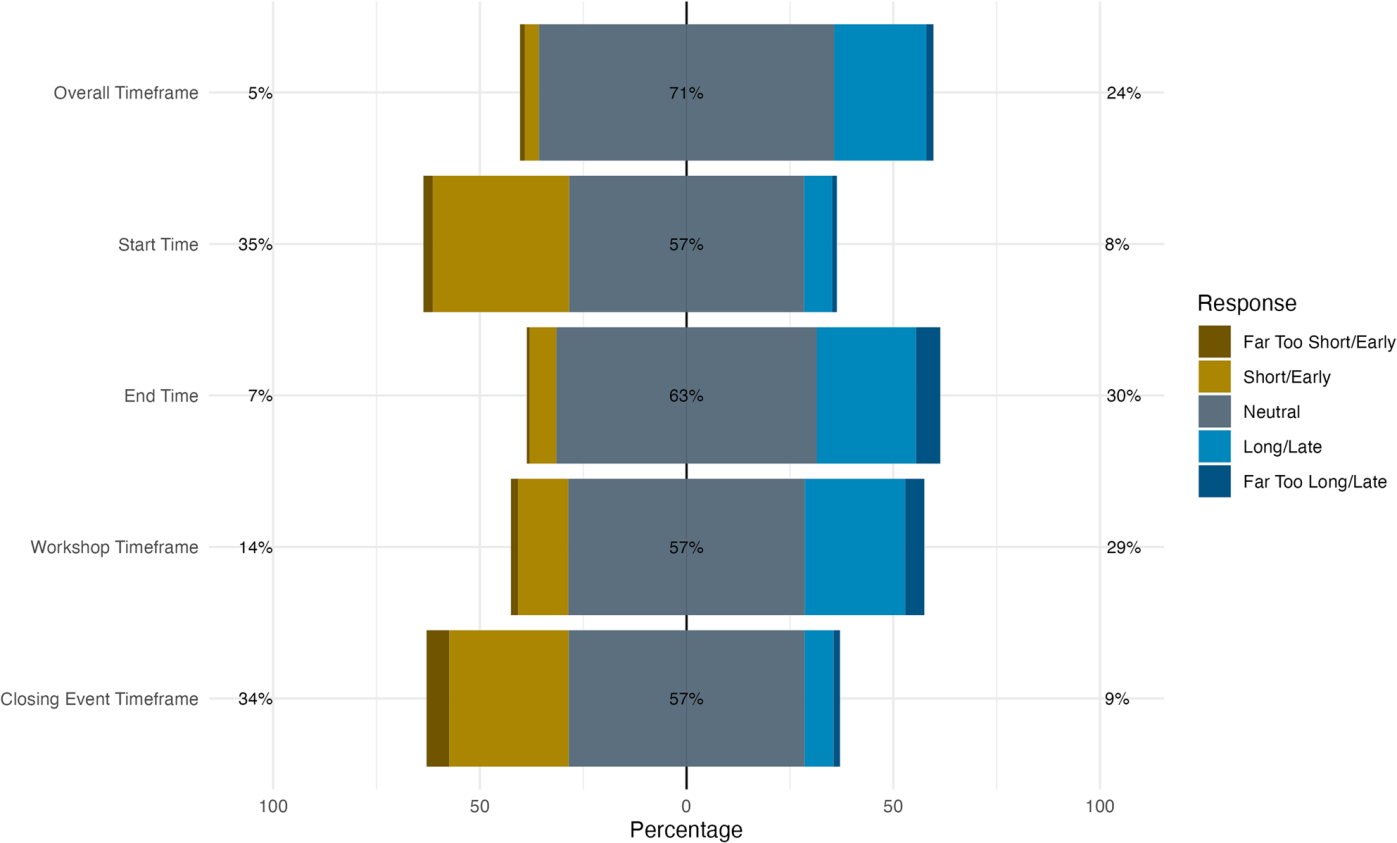
Distribution of perceived timing for various aspects of the event from the written evaluation. Bars show response distributions from early/short (left) to late/long (right). Left percentages aggregate "Far Too Short/Early" and "Short/Early" responses; right percentages aggregate "Far Too Long/Late" and "Long/Late". Centred percentage represents neutral responses

In the written evaluation, participants stated that they spent a median of 5 hours (Q25 – Q75: 4–7 hours) on the campus. Figure 2 shows participants' feedback on event timing. Most found the 5 pm to 3 am timeframe suitable. The 5 pm start was widely approved, with some voting for an earlier start - comparable to the 3 am end, which some found too late. For the workshop timeframe, over half deemed it appropriate. The 1 am to 3 am closing event garnered similar approval from just over half the respondents.

### Workshops

#### Amount of workshops

**Fig. 3:**
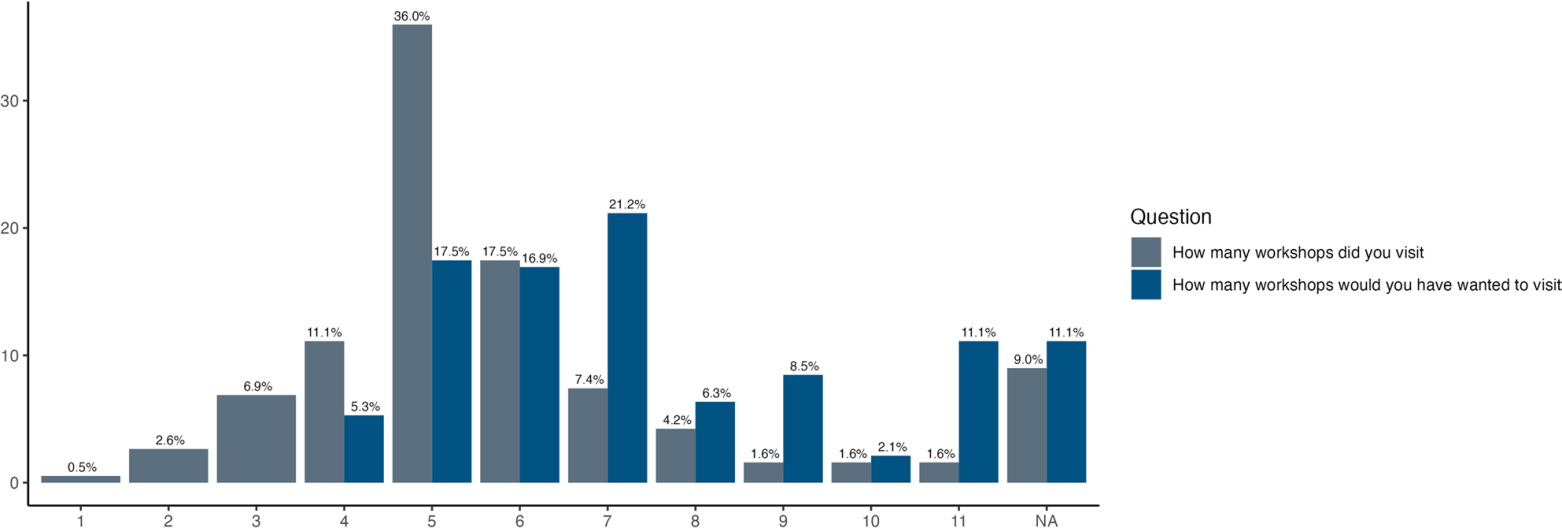
Feedback from the written evaluation on the number of workshops visited and the desired amount

A total of 1524 bookings for workshop timeslots were registered. Participants most commonly visited five workshops (Fig. 3). Most respondents, however, would have preferred to visit seven stations.

#### Duration of the workshops

**Fig. 4:**
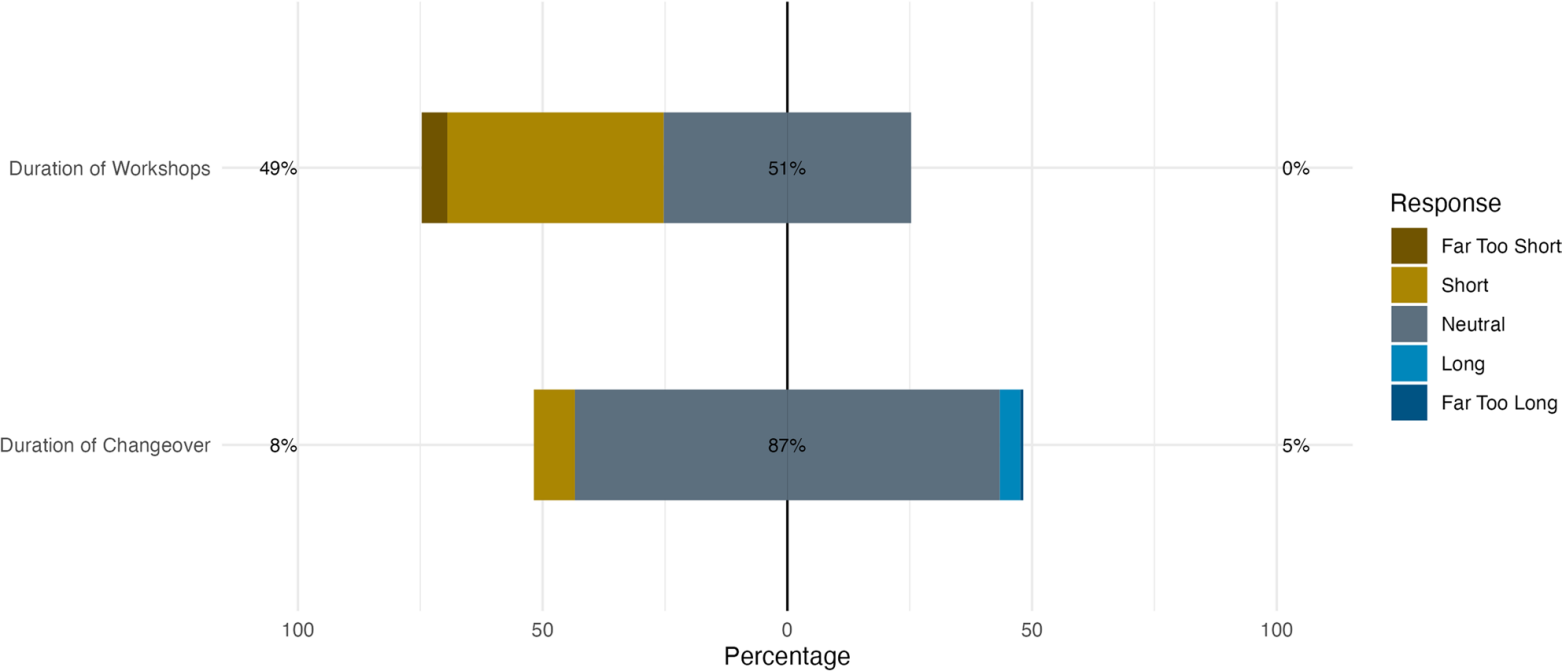
Distribution of perceived duration of workshops (30 min) and changeover period (15 min) from the written evaluation. Bars show response distributions from short (left) to long (right). Left percentages aggregate "Far Too Short" and "Short " responses; right percentages aggregate "Far Too Long" and "Long". Centred percentage represents “neutral” responses

For the duration of the workshops (30 min, Fig. 4), opinions were divided between appropriate and (far) too short. Regarding the changeover period (15 min), most respondents rated it as appropriate.

#### Subjective learning outcome and newly acquired skills

Most participants rated the event's general learning outcome positively, with 38.6% (N = 66) deeming it "very high" and 45.0% (N = 77) "high". 7.6% (N = 13) found it "neutral," 5.3% (N = 9) "low," and 1.2% (N = 2) "very low". 2.3% (N = 4) abstained from rating.

When asked which new skills were learned during the written evaluation, resuscitation, blood sampling, ultrasonography, central venous puncture, laparoscopy, puncturing, and medical interviewing were mentioned most often.

#### General improvement ideas for workshops

Qualitative analysis of workshop improvement ideas suggested expanding the variety of partaking disciplines and increasing the accessibility for lower semesters. Participants called for more physician involvement over student tutors, longer durations for some workshops, and increased workshop capacity overall. Better access control for limited-registration stations was recommended. Participants criticized the scarcity of workshops for nursing and midwifery students.

Workshop subjects

When asked which courses should definitely be offered again, laparoscopy, sonography, ENT, surgery, emergency medicine, and placing intravenous catheters were mentioned frequently. When asked which courses should not be offered again, most students replied that they liked all of them. However, microscopy, laparoscopy, and conversation training were mentioned individually. 

### Social program

#### Quantitative feedback on social program

**Fig. 5:**
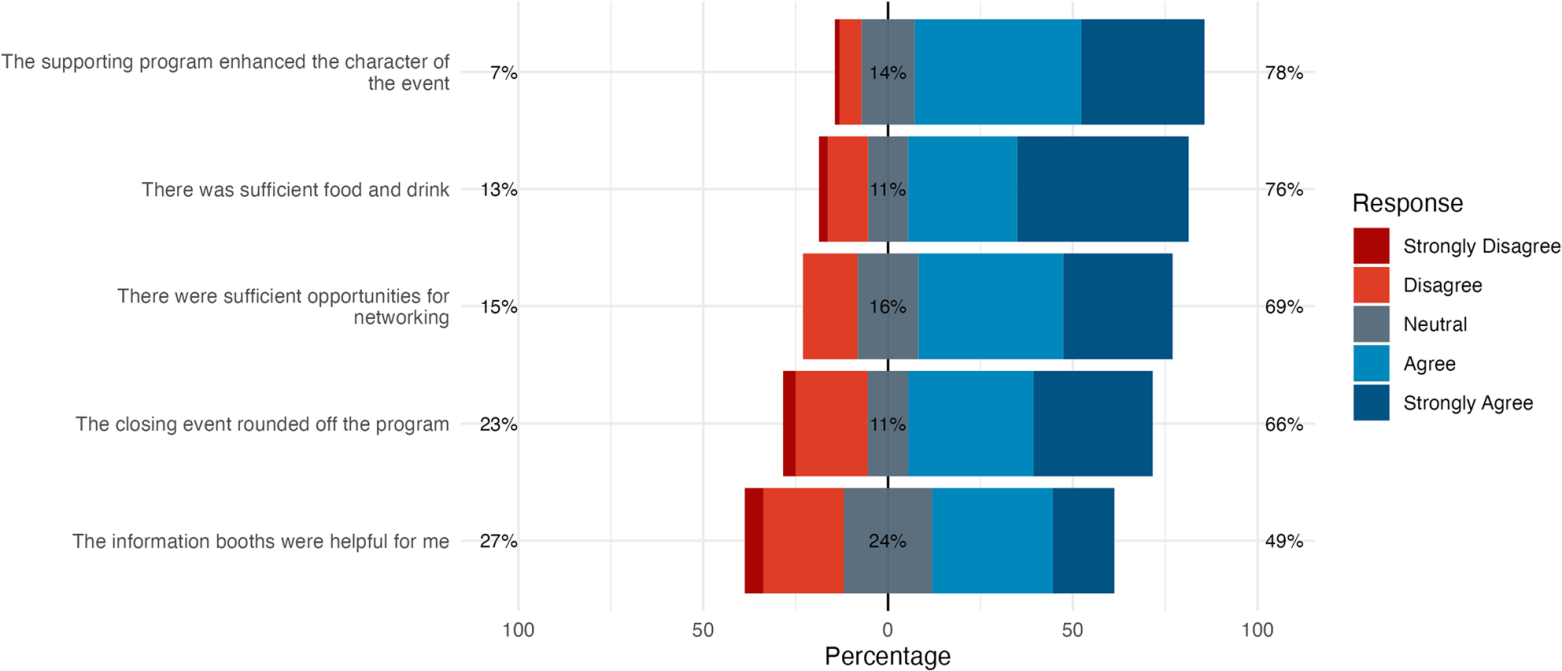
Distribution of agreement for various aspects of the social program from the written evaluation. Bars show response distributions strongly disagree (left) to strongly agree (right). Left percentages aggregate "Strongly Disagree" and "Disagree" responses; right percentages aggregate "Strongly Agree" and "Agree". Centred percentage represents neutral responses

Most participants felt the social program enhanced the event (Fig. 5). The satisfaction with food and drinks was high. Most participants reported sufficient chances to network. The closing event was well-received, effectively concluding the event for most. Feedback on information booths was more mixed.

### Qualitative feedback on social program

#### Qualitative analysis from written and oral student feedback

The qualitative analysis of interviews and written comments yielded several categories such as motivation, expectations, perceived benefits, networking, and suggestions for improvement (Table 2). To illustrate these themes, representative invivo quotes with participant codes are presented in Table 4.

**Table 4:** Themes from the qualitative analysis with illustrative participant quotes (translated from German; participant codes in brackets)

Theme	Quote and Participant
Motivation	“The first event after COVID – everyone just had this urge for practical work, right?“ [P30] “Cool thing, so you can really practice a little bit in a practical way. After Corona, where something like that kind of fell by the wayside.” [P33] “We thought it was a cool format to try out, to see what it’s like in smaller groups. … And we actually thought it was a nice idea to do it in the evening and in a relaxed atmosphere.” [P35] “Topics that sometimes get neglected in our studies and, on the other hand, the party.” [P46]
Expectations	“That you are taught skills that will actually be useful later on and are fun at the same time.” [P30] “Perhaps combining theory and practice well, simply trying something out for yourself that you would otherwise only hear about in theory.” [P30] “Just to gain some different insights and learn some new skills? Just to broaden your horizons a bit and meet new people.” [P39]
Expectations met	“Immediately impressed or convinced from the very first workshop.” [P33]
New contacts established	“You can make lots of contacts with other teachers and students.” [P29] „“If you want to do a clerkship, it means you already know someone you could approach.” [P30] “For example, we also went to a workshop in nursing science and just talked: how does their degree actually work, and so on? It was actually quite interesting to hear.” [P37]
Benefited in other ways	“I think it has a very high value for later professional practice.” [P29] “Because you also get a sense of whether something suits you or not – whether it’s fun or really not.” [P30] “Once you’ve seen it, I think you learn things in a completely different way than if you just learn them by heart.” [P33]
Preparation for clinic	“It gives a glimpse of what’s coming beyond the textbook. […] A great preparation for clerkships.” [P38] „Insight into the clinic.“ [P39] “I can imagine that you might get some inspiration, but that it’s not long-term or lasting. It’s not like permanent training.“ [P40]
Self-efficacy	“that you become more confident, that if you were to do it for the first time on a real patient, you wouldn’t be so nervous.“[TN30b; 00:03:12]
Positive feedback/requests	“I think it’s really cool that there are also interdisciplinary aspects, including nursing and midwifery sciences. I think that’s really cool. But I think it’s always neglected that you work on things together and later you work together as a team.” [P36]a

Students were very motivated because they longed for practice after theory and restrictions due to COVID. They also wanted to learn new hands-on skills and gain insights into new fields of work. In addition, students saw the event as a good preparation for the practical year, clinical clerkships and the practical exams. Furthermore, networking and social aspects also motivated participation.

In terms of expectations, learning new skills, gaining new insights, having fun while doing it, and making new contacts were mentioned. Some also came with no expectations at all. The majority of respondents indicated that expectations had been met or even exceeded, also mentioning an increased confidence.

Analysis showed most participants made valuable new contacts, especially for rotations, doctoral work, and practical years. Inter-semester and interdisciplinary connections were valued, though physician interactions could improve.

Participants found the event beneficial for clinical preparation, while recognizing the limitation of a single skill event. They appreciated field exposure and comprehensive event structure.

Furthermore, the participants stated that they benefited from the fact that they were able to experience different fields. Otherwise, the overall experience and the social aspect and the party were positively mentioned several times.

The overall feedback was very positive. The event was very well appreciated, and it was emphasized that the event should definitely be held again more often or on a regular basis. In addition, participants perceived the event as as well planned and organized. Especially the interdisciplinarity and the small groups and the good supervision were complimented.

### Overall rating

In summary, 96.3% of participants (*N* = 182) rated the event “very good” or “good”: 57.7% (*N* = 109) “very good” and 38.6% (*N* = 73) “good”. 3.2% (*N* = 6) found it satisfactory, 0.5% (*N* = 1) unsatisfactory. No one chose the neutral rating.

Of 189 respondents, 95.2% (*N* = 180) “definitely” agreed to repeat the event, with 3.2% (*N* = 6) selecting “rather yes”. Only 0.5% (*N* = 1) chose “definitely not”. Two participants (1.06%) abstained. No one selected neutral or “rather not”.

In total, 98.4% (*N* = 186) supported repeating the event.

## Discussion

This project report describes a medical education event that combined practical workshops with social and networking opportunities. In general, the event received very positive feedback, however, there are a few aspects that deserve discussion.

### Educational value

Regarding the “Night of Skills” as an educational intervention, potential benefits for participants, particularly in skill performance, should be discussed. Most participants rated their educational outcome highly, and many wished to attend more workshops than they could. Of note, the study did not assess actual skill performance, and data comprises only subjective judgments (Kirkpatrick level I) [32]. However, some educational values can be derived from these results:

From the perspective of experiential learning [28, 29], the “Night of Skills” offered exposure to multiple cycles of Kolb’s learning stages: brief instructor demonstrations and peer observation provided *concrete experience* and *reflective observation*, tutor input and links to prior teaching supported *abstract conceptualisation*, and supervised hands-on practice with feedback enabled *active experimentation* within each workshop. Informal discussions during breaks and the social programme likely extended these reflective processes beyond the individual stations. Moreover, the short, focused workshops with clearly defined learning objectives likely reduced cognitive load [33] within each session, while the elective format of the event enabled participants to self-regulate their cumulative cognitive load by deciding how many workshops to attend.

Although the event lacked repeated exposure or formal curricular integration, each session likely contributed to deliberate practice, which is crucial for performance improvement [15, 34]. Moreover, allowing participants to choose sessions promotes autonomy-supportive teaching, enhancing learner motivation [35, 36]. Motivation and feedback are key to educational outcomes, and the high participation of both students and instructors, even beyond regular hours, reflect a strong commitment [12, 36].

Many participants felt the event prepared them for clinical clerkships and practice, indicating a boost in self-efficacy. Hands-on experiences, crucial for building self-efficacy [37], likely contributed to this confidence. While the link between self-efficacy and learning outcomes is still being studied, some beneficial effects can be assumed [37]. Lastly, the event’s open, welcoming atmosphere and face-to-face interactions likely fostered a sense of belonging within the “Community of Practice” [30], supporting professional development.

### What can be learnt for possible future events and research?

The evaluation also offers insights for other institutions organizing similar events and for future iterations of the “Night of Skills” itself. Based on participants’ workshop choices and their feedback on the organisation and social programme, we derived concrete implications for the planning of similar events, which are summarised in Table 5. The overall timeframe (5 pm to 3 am) was well-received, though an earlier ending could be considered. The timing fits within the range of similar Skills-Lab events in Germany (Additional File 3). Advertising through peers and email remains key, though students expressed a need for a timely, broader communication.

**Table 5:** Main learnings from the “Night of Skills”

Aspect	Take-home points
Organisation and timeframe	- A Friday afternoon to night schedule (approximately 5–7 pm to 1–3 am) is appropriate. - Timely communication is essential, with primary advertising channels including email and social media. - A joint organizing committee with faculty and students is beneficial. - An easy-to-use online registration system can facilitate last-minute changes and cancellations
Workshops	- Suggested workshop duration is 45 min, with a 15-minute changeover. - Participants typically aim to attend 5–7 workshops. - Balance general and specific topics, as well as basic and advanced levels, to include beginning students and those from non-human medicine programs. - Maximize seating while keeping group sizes manageable. - Include a mix of faculty and peer instructors.
Social program	- Long events should incorporate a social program. - Allocate a dedicated, welcoming area with space for networking, food and drinks, and information booths. - A centralized closing event can enhance student engagement.
Other aspects	- Interdisciplinary workshops are well-received.

Regarding workshops, most students attended around five workshops and wished to attend more, but capacity limitations must balance with total participant numbers. With nearly 400 attendees, the event is likely among the largest of its kind. A wide range of workshop topics was offered, which was appreciated. While some advanced topics, like ENT surgery, were seen by a few as unnecessary, others valued them. A small number of advanced workshops can motivate students. Workshop duration of 30 min seemed short, so an extension to 45 min might be better, while the 15-minute changeover was reasonable.

The event’s social aspects were well-received, with participants valuing networking opportunities, catering, and the closing event. Food, drinks, and networking were especially popular, though more dedicated spaces for interactions is needed in future events. The student-organized closing event was positively reviewed and offers a good opportunity to conclude such an event, but should be limited to one area.

Beyond these practical implications, future iterations of the “Night of Skills” could benefit from a closer link to the formal curriculum, for example by aligning selected workshops with curricular activities to foster repeated learning. Targeted outreach strategies to increase interprofessional participation could further enhance the educational value of the event for all represented professions. As the format is scaled and more systematically integrated into the curriculum, accompanying research should include longitudinal, objective assessments of selected skills to better capture sustained competency gains.

### Comparison to similar events

To the authors’ knowledge, while similar event formats very likely exist in medical skills labs worldwide, peer-reviewed literature on comparable events is sparse. The closest comparable studies describe simulated night shifts where students work on multiple cases as a team (mimicking emergency department workflows) and report positive effects on self-efficacy, satisfaction, transition to residency or readiness for internships [38–41]. Given the limited data, the authors informally surveyed the German skills lab network (“SkillsLab-Forum”) to find comparable events within German-speaking regions. Results (Additional File 3) indicate many initiatives with a focus on simulated night shifts with 20–50 students, often interprofessional. In contrast, our “Night of Skills” stands out for its large scale (nearly 400 participants), a combination of skills training with a social program, and a wide variety of workshops.

### Limitations

There are several limitations to this approach. First, the data is from a single event at one institution, making it specific to this design and cohort. Second, although the event aimed for interdisciplinarity, over 90% of registrants were from the human medicine program. While this mirrors the faculty’s student distribution, more students from other programs should be involved in future events to enhance interdisciplinary participation. Third, only self-reported data without objective skill assessments was collected, so benefits are inferred from the data and educational assumptions. In particular, high ratings may partly reflect enthusiasm or novelty effects rather than true gains in clinical competence. Fourth, the evaluation was conducted just after the event, without follow-up on long-term perceptions. This is particularly important as future studies should complement self-ratings with objective assessments of skill performance both directly after the event and at later follow-up time points. Fifth, due to the survey response rate, non-response bias cannot be ruled out, as non-respondents might have held different views than those who completed the evaluation. Finally, there may be a general selection bias, as only highly motivated students who voluntarily registered attended the event. Despite these limitations, given the scarcity of data on such events, the comprehensive evaluation offers valuable insights for other faculties planning similar programs.

## Conclusions

Junior doctors often feel not prepared for work [42], and events like the “Night of Skills” demonstrate a promising model for supplementing traditional medical curricula with intensive, practice-oriented experiences. Its success in combining practical skill development with networking opportunities addresses multiple facets of medical education and could ease the transition from university to clinic [43]. Future iterations and similar events at other institutions could significantly contribute to preparing health professions students for the complexities of clinical practice.

## Supplementary Information

Supplementary Material 1: The translated version of the interview guide.

Supplementary Material 2: The full numeric dataset of the quantitative data.

Supplementary Material 3: A table of skills events in german skills labs.

## Data Availability

The datasets supporting the conclusions of this article are included within the article and its additional files.

## Abbreviations

CBME: Competency-based medical education
CPR: Cardiopulmonary Resuscitation
CVC: Central venous catheterization
ENT: Ear, Nose, and Throat
NA: Not Applicable
Q25 – Q75: 25th and 75th percentiles
