# Supplementary material for: A faculty-wide “Night of Skills” to (not only) train medical skills: comprehensive evaluation study
Source: BMC Med Educ. 2026 May 9;26:742. doi: 10.1186/s12909-026-09066-1 (PMC13156877; doi:10.1186/s12909-026-09066-1)
Supplement: Supplementary file 2 — Supplementary Material 2: The full numeric dataset of the quantitative data. [file 12909_2026_9066_MOESM2_ESM.docx]

| Place | Skills-Lab or University Name | Name of Event | Event Time | Particpants  N | Participants  Characteristics | Educational Focus and Aspects | Ref. |
| --- | --- | --- | --- | --- | --- | --- | --- |
| Aachen | AIXTRA | NightShift | 3:45 pm – 4 am | - | 48 medical students + 16 paramedic students | Simulated hospital night shift | [1,2] |
| Berlin | Lernzentrum Charité | Nachtdienst | 10 pm – late | 35 (first come, first served) | Medical faculty (10th semester and above) | Simulated night shift in an emergency room. | [3,4] |
| Bonn | SkillsLab | Skills Night | 10 pm – 2 am | 12 - 20 | Medical faculty | Simulated night in an emergency room, focus on practical and theoretical skills in emergency situations, online evaluations comparing pre/post-COVID cohorts | [5] |
| Erlangen | PERLE | SkillsNight | 6 pm – 3 am | 6 | Interprofessional (50% human medicine, 50% nursing) | Simulated night shift in emergency medicine | [6] |
| Essen | SkillsLab | Nachtschicht | - | - | - | emergency department, trauma room, and pre-hospital scenarios | [7] |
| Freiburg | StudiTZ | SkillsNight | 8 pm – 3 am | 25 | Interprofessional, medical faculty | Simulated emergencies with interdisciplinary medical cases, team-based problem-solving, and practical skill training (ZVK, Thoraxdrainage, Sonography, etc.) | [8] |
| Göttingen | STÄPS | - | 5 pm – 11 pm | 18 | Medical Students | Simulated night shift, based on peer assisted learning | [9] |
| Innsbruck | Med Uni Innsbruck | Skills Night | 6 pm – 4 am | 48 | Interprofessional (50% human medicine, 50% nursing) | Simulated night shift in emerngency medicvine with theoretical lessons before event | [10] |
| Köln | Kölner Interprofessionelles Skills Lab (KISS) | Eine Nacht im KISS | 2-hour shifts, 17:00–01:00 | 36 | Medical faculty, nursing | Students manage emergency department cases with real medical personnel, fire department, and professional actors. Focus on interprofessional teamwork in emergency settings. | [11,12] |
| Magdeburg | MAMBA | Trauma Night | 6 pm – late | Varied, semester-based | Medical students and faculty | Focus on trauma management and emergency medicine skills | [13] |

References:

1. Rische P. SKILLQUBE unterstützt die Nightshift (WS 23/24) in Aachen! Ski GmbH. 2024. Available from: https://de.skillqube.com/2024/02/26/skillqube-unterstuetzt-die-nightshift-ws-23-24-in-aachen/ [accessed Sep 17, 2024]

2. Arbeitskreis Notfallmedizin – Fachschaft Medizin RWTH Aachen. Available from: https://www.fsmed-aachen.de/die-fachschaft/projekte/arbeitskreis-notfallmedizin/ [accessed Sep 17, 2024]

3. Dohle NJ, Franz H, Gräf J, Bossert E, Buchmann M, Machner M, Penders D. Tag- oder Nachtdienst? Interprofessionelle studentische Simulationen gemeinsam planen. German Medical Science GMS Publishing House; 2023. p. DocP1.4. doi: 10.3205/22isls07

4. Hiß J. Nachtdienst. Lernzentrum Charité. Available from: https://lernzentrum.charite.de/projekte/nachtdienst/ [accessed Sep 15, 2024]

5. Michlmayr N, Knecht R, Brück A, Fuchs M-L, Protte D. Wiederbelebung der SkillsNight. Int Ski-Symp 2023 Köln, Germany: Uniklinik Bonn; 2023. Available from: https://gesellschaft-medizinische-ausbildung.org/files/Ausschuesse/Praktische_Fertigkeiten/17_Koeln_iSLS2023_-_Programm_Stand_2023_03_16.pdf

6. Kursangebot SkillsLab PERLE, Medizinische Fakultät, Friedrich-Alexander-Universität Erlangen. Med Fak. Available from: https://www.med.fau.de/studium/medizin/skills-lab-perle/kursangebot/ [accessed Sep 15, 2024]

7. SkillsLab Essen auf Instagram: “Recap Nachtschicht🌛 Unsere Nachtschicht war ein voller Erfolg! Die Teilnehmer:innen konnten in der Notaufnahme, im Schockraum oder präklinisch in verschiedenen Fallbeispielen die Schauspielpatient:innen versorgen. Ein riesiges Dankeschön an @feuerwehr_essen für die Unterstützung und das gemeinsame Lernen. Wir wünschen allen Azubis viel Erfolg für das Examen🍀 Es hat uns dem Skillslab-Team unglaublich viel Spaß gemacht und freue uns über das bisherige Feedback❣️ Wir freuen uns auf die nächste Nachtschicht mit euch😊.” Instagram. 2024. Available from: https://www.instagram.com/p/C2YBqiQNrSZ/ [accessed Sep 17, 2024]

8. STUDITZ - Skillslab der Albert-Ludwigs-Universität Freiburg | Aktivitäten. Available from: https://mdek-web.ukl.uni-freiburg.de/skillslab/index.php?site=aktiv [accessed Sep 15, 2024]

9. Feige MF, An der Brügge K, Sandrock LJ, Szallies HR, Hubricht D. Studentische Simulation einer Nachtschicht in der Notaufnahme. German Medical Science GMS Publishing House; 2024. p. DocV2.1. doi: 10.3205/24isls07

10. Video: Das war die “Skills Night 2024” - myPoint. Available from: https://www.i-med.ac.at/mypoint/news/783563.html [accessed Sep 15, 2024]

11. Eine Nacht im Kiss. Available from: https://medfak.uni-koeln.de/studium-lehre/kiss-skills-lab/eine-nacht-im-kiss [accessed Sep 16, 2024]

12. van Edig M, Pickert L, Bornemann S, Brinkmann J, Bußhoff J, Struck T, Stosch C. „Eine Nacht im Kiss" – eine Konzeptvorstellung. Neuruppin, Brandenburg, Germany; 2019.

13. Brinkema H, Riedel JN, Pietz C, Schelp A, Leschowski N. AG Notfallmedizin im MAMBA Skillslab. German Medical Science GMS Publishing House; 2019. p. DocP02-08. doi: 10.3205/19isls064
