# Supplementary material for: A faculty-wide “Night of Skills” to (not only) train medical skills: comprehensive evaluation study
Source: BMC Med Educ. 2026 May 9;26:742. doi: 10.1186/s12909-026-09066-1 (PMC13156877; doi:10.1186/s12909-026-09066-1)
Supplement: Supplementary file 3 — Supplementary Material 3: A table of skills events in german skills labs. [file 12909_2026_9066_MOESM3_ESM.docx]

**Research Question:** What do participants expect from the event and how do they benefit from it?

Thank you for agreeing to answer some questions about this evening. The goal of this survey is to evaluate expectations, motivation, and the benefits of this event.

First, I would be interested in knowing in what capacity you are attending today.

- **Student:** Which subject? Which semester?
- **Tutor:** Which subject? Which semester?
- **Instructor:** Which subject?
- **Staff:** Which department?

What motivated you to participate in this event?

What expectations did you have for this event, or what did you hope to gain from this evening?

Were these expectations met?
Yes No
If yes or no, how so?

Do you feel you were able to learn new skills here? If yes, what are they?

Were you able to make new connections with fellow students, colleagues, or instructors? If yes, with whom and in what way?

How else have you benefited from this event?

Do you feel better prepared for the clinical environment after attending this event? If yes, how so?

What did you miss or what would you like to see in future events?
